# Comparison between the mesenteric fixation method (MEFIX) and conventional methods at preventing the occurrence of Petersen’s hernia: a study protocol for a multicenter randomized controlled trial

**DOI:** 10.1186/s13063-023-07841-9

**Published:** 2024-01-02

**Authors:** Jae Kyun Park, Dae Hwan Kim, Tae-Yong Jeon, Sang-Ho Jeong, Tae Han Kim, Jae-Seok Min, Rock Bum Kim, Young Joon Lee, Ji Ho Park, Young Gil Son, Ki Young Yoon, Kyung Won Seo, Ki Hyun Kim, Yoonhong Kim, Hyun Dong Chae, Sun Hwi Hwang, Si-Hak Lee, Jae Hun Chung, Hyoung-Il Kim, Dong Jin Park, Kwang Hee Kim, Sang Hyuk Seo, Sung Jin Oh, Woo Yong Lee, Chang In Choi

**Affiliations:** 1 Department of Surgery, Pusan National University Hospital, Pusan National University School of Medicine, Biomedical Institution, Busan, 49241 Republic of Korea; 2 Department of Surgery, Gyeongsang National University College of Medicine and Gyeongsang National University Changwon Hospital, Changwon, Republic of Korea; 3 Department of Surgery, Dongnam Institute of Radiological and Medical Sciences, Cancer Center, Busan, Republic of Korea; 4 Regional Cardiocerebrovascular Disease Center, Gyeongsang National University Hospital, Jinju, Republic of Korea; 5 Department of Surgery, Gyeongsang National University College of Medicine and Gyeongsang National University Hospital, Jinju, Republic of Korea; 6 Department of Surgery, Keimyung University Dongsan Hospital, Daegu, Republic of Korea; 7 Department of Surgery, Kosin University College of Medicine, Busan, Republic of Korea; 8 Department of Surgery, School of Medicine, Catholic University of Daegu, Daegu, Republic of Korea; 9 Department of Surgery, Pusan National University Yangsan Hospital, Pusan National University School of Medicine, Yangsan, Republic of Korea; 10 Department of Surgery, Yonsei University College of Medicine, Seoul, Republic of Korea; 11 Department of Surgery, Ulsan University Hospital, University of Ulsan College of Medicine, Ulsan, Republic of Korea; 12 Department of Surgery, Inje University Busan Paik Hospital, Busan, Republic of Korea; 13 Department of Surgery, Inje University Haeundae Paik Hospital , Busan, Republic of Korea

**Keywords:** Internal hernia, Minimally invasive surgery, Gastrectomy, Gastric neoplasm

## Abstract

**Background:**

Petersen’s hernia, which occurs after Billroth-II (B-II) or Roux-en-Y (REY) anastomosis, can be reduced by defect closure. This study aims to compare the incidence of bowel obstruction above Clavien–Dindo classification grade III due to Petersen’s hernia between the mesenteric fixation method and the conventional methods after laparoscopic or robotic gastrectomy.

**Methods:**

This study was designed as prospective, single-blind, non-inferiority randomized controlled multicenter trial in Korea. Patients with histologically diagnosed gastric cancer of clinical stages I, II, or III who underwent B-II or REY anastomosis after laparoscopic or robotic gastrectomy are enrolled in this study. Participants who meet the inclusion criteria are randomly assigned to two groups: a CLOSURE group that underwent conventional Petersen’s defect closure method and a MEFIX group that underwent the mesenteric fixation method. The primary endpoint is the number of patients who underwent surgery for bowel obstruction caused by Petersen’s hernia within 3 years after laparoscopic or robotic gastrectomy.

**Discussion:**

This trial is expected to provide high-level evidence showing that the MEFIX method can quickly and easily close Petersen’s defect without increased postoperative complications compared to the conventional method.

**Trial registration:**

ClinicalTrials.gov NCT05105360. Registered on November 3, 2021.

**Supplementary Information:**

The online version contains supplementary material available at 10.1186/s13063-023-07841-9.

## Background

Petersen’s hernia was first described in 1900 by Walther Petersen, a German surgeon, following the appearance of an internal hernia after gastrectomy and gastrojejunostomy (GJ) [1]. The space between the mesentery of the small intestine and the transverse colon after the gastrojejunal or esophageal-jejunal anastomosis is called “Petersen’s defect.” In Petersen’s hernia, the mesentery rotates and twists; therefore, an emergency event may occur in which the entire small intestine is necrotic due to impaired blood circulation.

After laparoscopy-assisted distal gastrectomy was first performed in 1994, it has become the standard surgery for early gastric cancer, as reported by Kitano et al. [2]. It is minimally invasive compared with open surgery, which reduces the risk of postoperative adhesions, recovery time of bowel movements, hospital stay, and postoperative pain. However, due to relatively fewer adhesions, the incidence of internal hernias is higher in laparoscopic surgery than with open surgery, and the incidence of Petersen’s hernia after laparoscopic REY gastrojejunostomy is reported to be 1.7–9.7% [3–8]. The anti-adhesive agent, which has been widely used recently, is also thought to be one of the reasons for the increased incidence of internal herniation [9]. According to Blockhuys et al., the incidence of internal hernias decreases when Petersen’s defect closes [10]. Many surgeons also believe that closure of the mesenteric defect with nonabsorbable sutures prevents Petersen’s hernia.

As the method of Petersen’s defect closure, the mesentery of the small intestine and transverse colon is closed with a suture. However, in patients with thin mesentery, tearing is common, and there is a high risk of damage to the small arterioles, which can lead to bleeding and ischemia of the small intestine. Additionally, the narrow surgical field and the close proximity of the surgical instruments can make the suturing of the mesentery challenging. The MEFIX technique is a recently developed approach for which there have yet to be any comparative studies on operative time with the conventional defect closure method. In our previous retrospective study, we exclusively compared this technique, and the time taken to close Petersen’s defect was 3.7 ± 1.1 min for the MEFIX method and 7.5 ± 1.5 min for the conventional method, respectively (*p* < 0.001) [11]. It suggests that a more efficient technique could be necessary for closure of the Petersen’s defect.

**Fig. 1:**
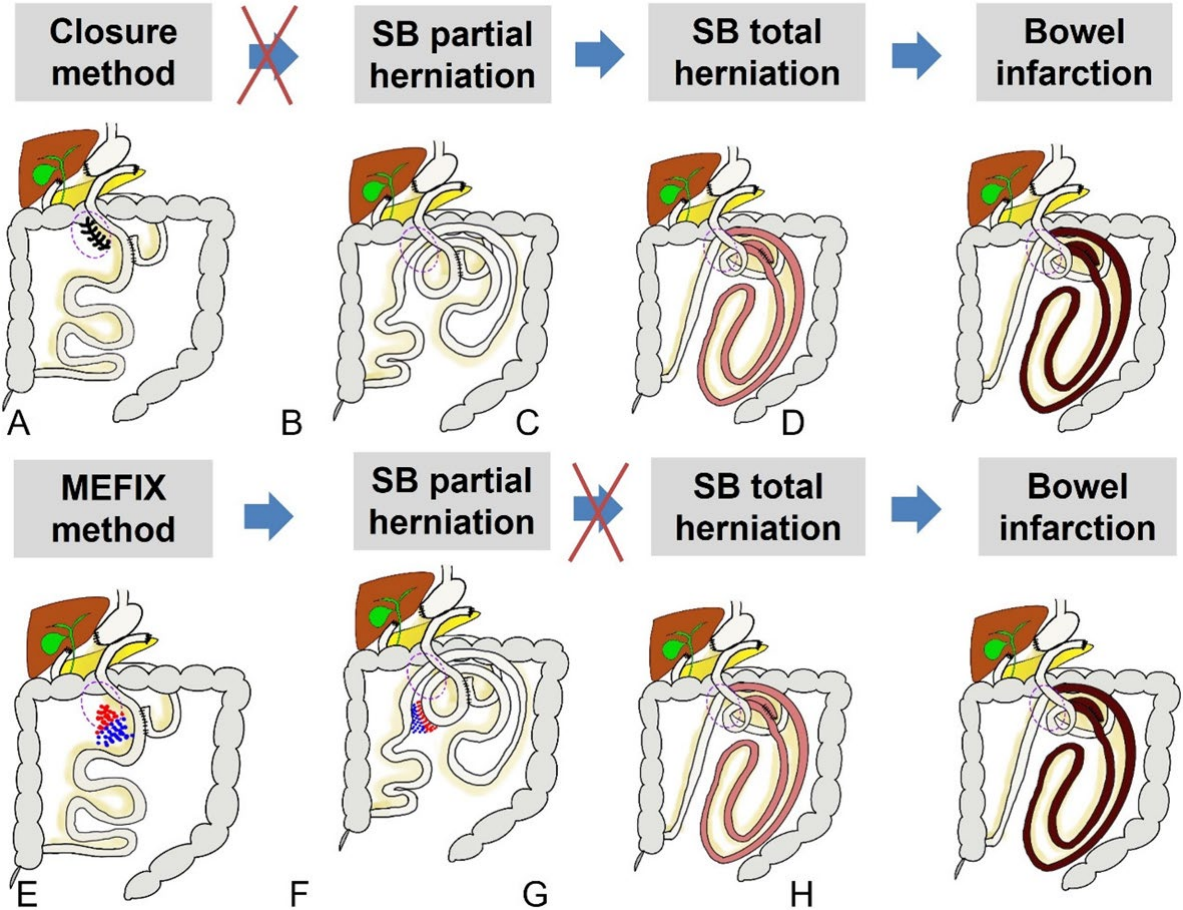
The concept of conventional closure (**A**–**D**) and mesentery fixation methods (**E**–**H**). This method allowed partial herniation of the bowel, but it prevented of total SB herniation and necrosis. MEFIX, mesentery fixation; SB, small bowel

In this study, we propose a novel mesenteric fixation method (MEFIX) to prevent Petersen’s hernia, in which a portion of the mesentery of the small intestine is fixed to the mesentery of the transverse colon. It prevents Petersen’s hernia by preventing total herniation of the mesentery of the small intestine even if partial internal hernia is possible by creating the effect of adhesion of the small intestine to the surgical site during laparotomy [11] (Fig. 1).

## Methods

The trial protocol is identified as version 2.0 of 27 December 2022. This protocol has been drafted in accordance with the Standard Protocol Items: Recommendations for Interventional Trials (SPIRIT). The SPIRIT checklists can be found as Additional file 1.

### Objectives

This study aims to compare the incidence of intestinal obstruction above the Clavien-Dindo classification grade III due to Petersen’s hernia between the MEFIX method and conventional closure methods after minimally invasive surgery such as laparoscopic or robotic gastrectomy.

### Study design and participants

**Fig. 2:**
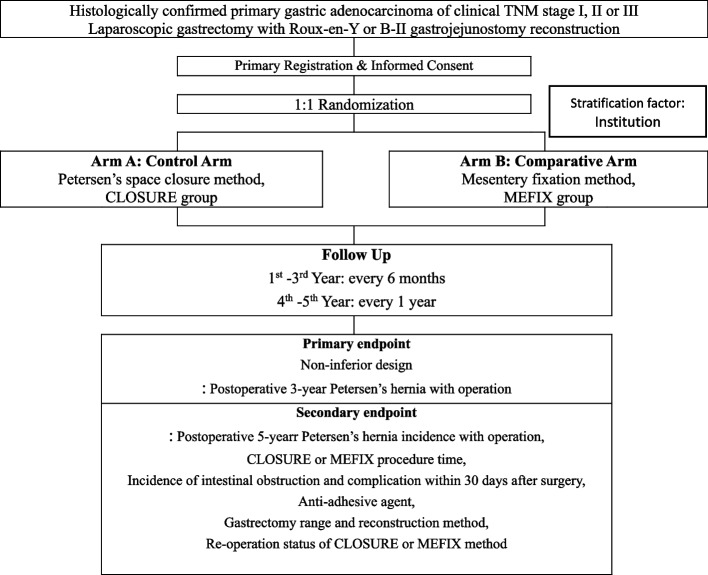
Flow chart of study protocol

This study was designed as prospective, single-blind, non-inferiority randomized controlled multicenter trial in Korea. We enroll patients with histologically diagnosed gastric cancer of clinical stages I, II, or III who underwent B-II or REY anastomosis after laparoscopic or robotic gastrectomy. Patients who meet the inclusion criteria are randomly assigned to two groups: a CLOSURE group that underwent conventional Petersen’s defect closure and a MEFIX group that underwent the mesenteric fixation method. Computed tomography (CT) will be performed every 6 months for the first 3 years after surgery and every 1 year for the next 2 years. A history of outpatient or emergency department visits for symptoms of internal hernia, such as vomiting and abdominal pain, or emergency surgery for internal hernia will be examined (Fig. 2 and Table 1).

**Table 1:** Timetable of the study period

	Study period										
	Enrolment	Allocation	Post-allocation (months)								Close-out
Time point		Surgery	6	12	18	24	30	36	48	60	5 years after surgery
Enrolment											
Eligibility screen	x										
Informed consent	x										
Allocation		x									
Interventions											
CLOSURE		x									
MEFIX		x									
Assessment											
Evaluation of Petersen’s hernia			x	x	x	x	x	x	x	x	x

### Eligibility criteria

#### A) Inclusion criteria

- Histologically confirmed primary gastric adenocarcinoma
- No evidence of other distant metastasis
- Underwent laparoscopic total or distal gastrectomy
- Underwent robotic total or distal gastrectomy
- Reconstructed by B-II or REY procedure
- Aged 20 or older
- Patients with appropriate activity conditions: 0 or 1 on the Eastern Cooperative Oncology Group (ECOG) scale
- Patients who had not previously received abdominal chemotherapy or radiation treatment
- Patients who signed the consent form

#### B) Exclusion criteria

- Active double cancer (i.e., synchronous and metachronous double cancer within 5 disease-free years) and carcinoma in situ (i.e., lesions suggestive of intraepithelial or intramucosal cancer)
- Gastric cancer recurrence
- A history of abdominal surgery, with the exception of laparoscopic appendectomy, gallbladder resection, laparoscopic gynecologic surgery for benign neoplasms, and cesarean section
- Patients in whom other organs must be coordinated during the preoperative examination (however, laparoscopic cholecystectomy is included in the selection criteria due to gallbladder polyps and gallbladder disease)
- Pregnant or lactating women
- Diagnosed with mental illness in the medical records
- Patients taking whole-body corticosteroids, including herbal medicines
- An uncontrolled history of angina or myocardial infarction within 6 months before the study period
- Uncontrolled hypertension
- Patients suffering from a severe respiratory disease requiring continuous oxygen therapy

#### C) Elimination criteria

- If a patient has expressed an intention to discontinue participation in the study before or after the study has begun
- Participants who did not undergo an appropriate operation (laparoscopic or robotic distal gastrectomy with B-II or REY anastomosis) or do not have available outcome data

### Recruitment

We elucidate the details of the study to patients during their outpatient visits, concurrently verifying their interest in participation. Simultaneously, we also recruit potential study participants publicly within the institution through informational posters. The clinical researcher or authorized personnel should be able to explain the purpose and treatment of the test and show that they are aware of the benefits and risks of the test in this process. Participants should also be aware that they are required to join voluntarily and maintain the liberty to withdraw from the research at any time. The participants should have enough time to think about their participation before the informed consent form sign and discuss it with their families. The documentation of the discussion and the date of the prior consent shall be recorded in the source documentation. Subjects must write a written consent form. If the hospital that manages the subject’s informed consent does not have the same guidelines, the guidelines that manage the informed consent should be followed equally in all hospitals.

### Sample size

Sample size calculation was based on an assumed proportion of Petersen’s hernia of approximately 1.15% for the conventional close method from our previously published papers [11]. The estimated difference in Petersen’s hernia proportions between close and MEFIX methods was assumed as 0.0% based on the previous similar study [11]. Thus, we set the inferiority threshold at 3% for clinical relevance. Therefore, if the proportion of Petersen’s hernia of the MEFIX method is less than 4.15%, the MEFIX method is not considered inferior to the conventional close method.

A sample size of 199 per arm is required for a proportion test with a one-sided significance level of 0.025 and power of 80% with no difference of hernia proportion between the two methods and a non-inferiority margin for the MEFIX method of 3% (non-inferiority limit = 0.03, treatment A (experimental) group = 0.0115, treatment B (control) group = 0.0115). Further, we decided to increase the sample size by 10% to cover possible dropouts. Consequently, 222 participants were included per group, and a total of 444 were needed. This calculation was performed as the PASS 13 (Power Analysis and Sample Size Software (2014). NCSS, LLC. Kaysville, UT, USA).

### Statistical analysis

All outcomes are assessed using an intention-to-treat analysis that included all patients who were randomly assigned. However, patients who fall under “failure of major entry criteria,” such as those who are not treated at all or do not have available data after randomization, can be excluded from the ITT analysis. Per-protocol analysis also will be performed to evaluate the treatment effect under full compliance.

Demographic data and baseline characteristics of all randomized participants will be pooled. Data will be analyzed using R version 4.2.1 (R: a language and environment for statistical computing, R Foundation for Statistical Computing, Vienna, Austria). Continuous variables will be presented as mean and standard deviation or median and interquartile range. Categorical variables will be described as frequencies and percentages. We will analyze clinicopathological data and postoperative outcomes, including the primary and secondary endpoints, using the chi-square test, Fisher’s exact test, Student’s *t*-test (two samples), or Mann–Whitney *U*-test as appropriate methods based on the type of variables.

To assess the non-inferiority of the MEFIX method compared with the conventional close method, a bilateral 95% confidence interval (CI) of the absolute difference in the Petersen’s hernia rate between the MEFIX group and the CLOSURE group will be estimated. The upper bound of this CI will be compared with the 3% of non-inferiority margin. The primary analysis of the primary endpoint (proportions of bowel obstruction caused by Petersen’s hernia) is performed based on chi-square test without adjustment for imbalance in baseline covariates. And we will calculate the hazard ratio of MEFIX group compared to CLOSURE group by Cox proportional hazard analysis with adjusting covariates. Additionally, secondary endpoints will be analyzed with equivalent test, and dichotomous variables will be compared between the two groups using adjusted and unadjusted hazard ratios and 95% CI by Cox proportional hazard analysis. Unadjusted analyses of time to event for primary and secondary endpoints will be presented using Kaplan-Meier curves and analyzed for non-inferiority of median time to event using the log-rank test. Candidate variables will be subsequently incorporated into a logistic regression analysis. We will express multivariate comparisons as hazard ratios with corresponding 95% CI. For all analyses, *p* < 0.05 will be considered significant statistically. There will be no imputation used to handle missing data during the analysis.

### Plans to give access to the full protocol, participant-level data, and statistical code

The datasets and statistical code utilized in the current study, as well as the full protocol, are accessible from the corresponding author upon reasonable request.

### Randomization

Patients are randomly assigned in a 1:1 ratio to the MEFIX or CLOSURE group. Allocation is conducted by computer-generated permuted random blocks (size 4) stratified by 12 hospitals. Block randomization is performed by an independent biomedical statistician using R software. A principal investigator (PI) retains the random assignment tables from each hospital. When eligible patients are recruited from each hospital, the clinical research coordinator (CRC) at the PI’s institution is notified. The CRC then confirms the randomization result on the day prior to the surgery and subsequently informs the respective institution.

### Intervention description

The gastric cancer is diagnosed by preoperative biopsy, endoscopic ultrasonography, or CT. Laparoscopic or robot gastrectomy is performed. Partial omentectomy and D1+ lymph node resection are performed for early gastric cancer (EGC), while total omentectomy and D2 lymph node resection are performed for advanced gastric cancer (AGC). The lymph node resection extent follows the Japanese Classification of Gastric Carcinoma published in 2020 [12]. B-II or REY reconstruction is performed using intracorporeal and antecolic methods. Isoperistaltic or antiperistaltic anastomosis is performed differently depending on the preference of the technician. All technicians send surgical videos to the principal investigator (PI) before patient registration to verify quality control.

#### A) Operative methods for Petersen’s defect closure (CLOSURE group)

**Fig 3:**
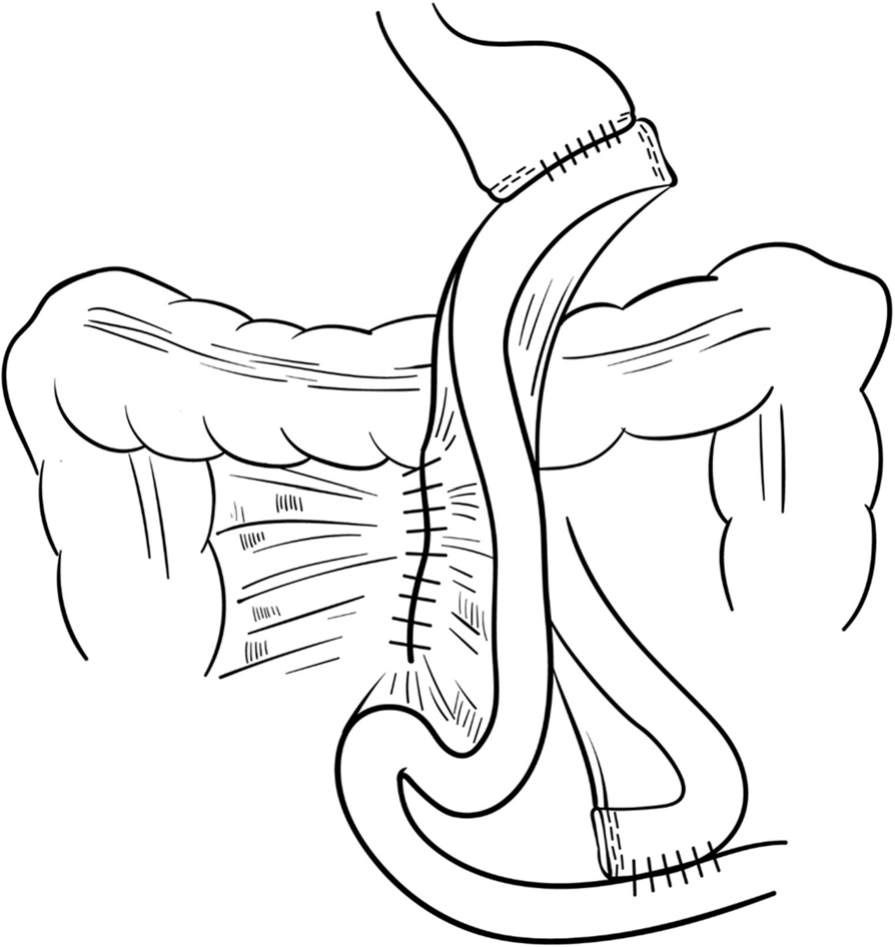
Petersen’s defect closure; CLOSURE group. Petersen’s defect was closed continuously using a nonabsorbable barbed suture between the jejunum and transverse colon

The Petersen’s defect, which occurs between the mesentery of the jejunum and transverse colon, is sutured with a nonabsorbable thread after a gastrojejunal or esophageal-jejunal anastomosis. First, a traction suture is performed between the jejunum and transverse colon, and Petersen’s defect is closed continuously using a nonabsorbable barbed suture V-Loc™ 3-0 (Medtronic VR, Minneapolis, MN, USA). Because leaving the tip of the barbed suture can cause bowel obstruction, backward suture with the V-Loc 3-0 was performed two to three times at the last suturing step. Subsequently, the tip is cut short after anchoring (Fig. 3).

#### B) Operative methods for mesentery fixation (MEFIX group)

The MEFIX method, by fixing the jejunal mesentery to the transverse mesocolon, allows for small bowel herniation. However, it also helps prevent whole bowel ischemia by inhibiting the twisting of the small bowel mesentery.

**Fig. 4:**
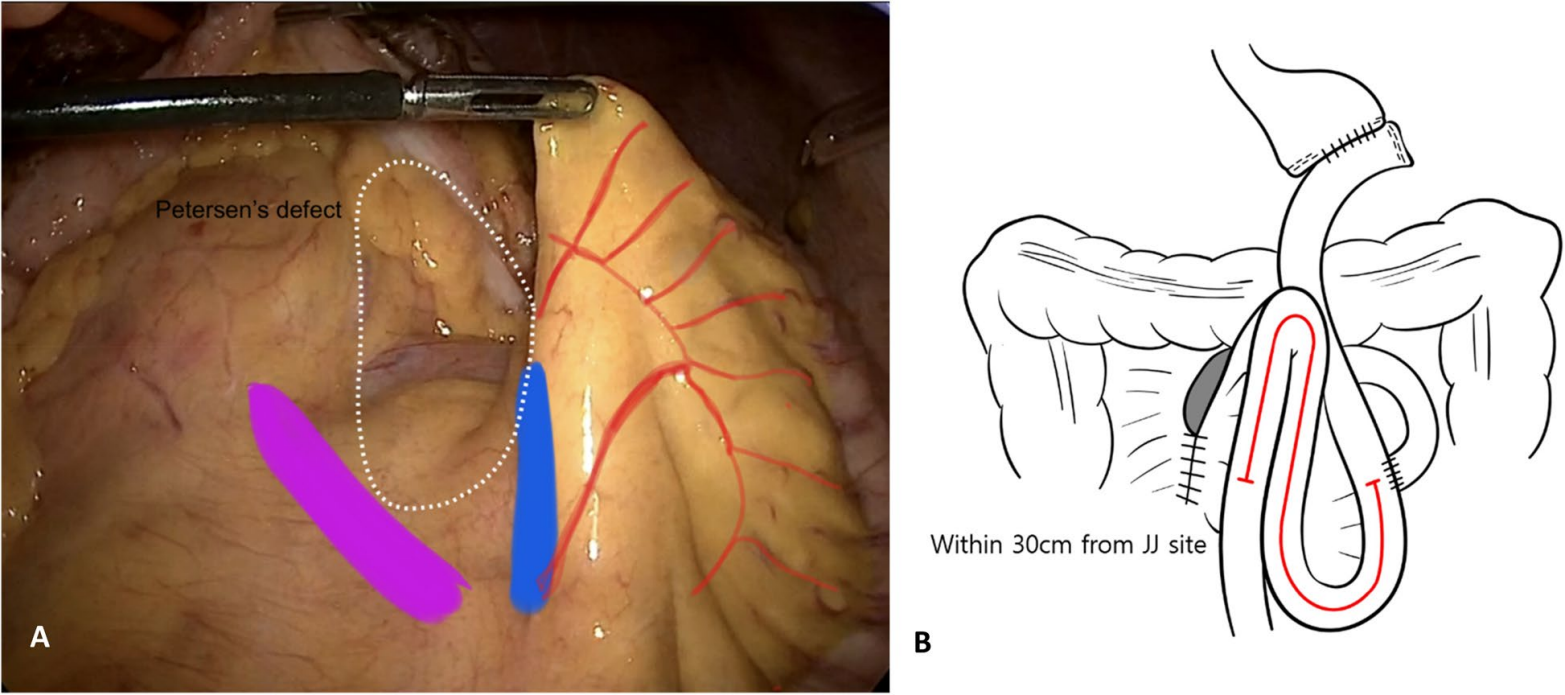
**A** Jejunal mesentery side (blue area) and transverse mesocolon (purple area). Petersen’s defect is outlined in the dotted area. **B** Petersen’s defect closure; MEFIX group. The jejunal mesentery was fixed to the transverse mesocolon using a nonabsorbable barbed suture. JJ, jejunojejunostomy

The arcade artery (blue area, jejunal side) and transverse mesocolon (purple area, colonic side) have relatively few blood vessels under the vasa recta of the jejunal mesentery within 30 cm distal to the jejunojejunostomy site (Fig. 4a). A traction suture is performed at the suture origin, and the mesentery of the jejunum is fixed to the transverse mesocolon with a nonabsorbable barbed V-Loc™ 3-0 suture (Medtronic VR, Minneapolis, MN, USA). It is sutured from the inferior arcade artery to the mesenteric root, which is present in approximately 2/3 of the mesentery of the small intestine. Similarly, in the final suturing step, the reverse suture is performed two to three times with the V-Loc 3-0, and the tip is cut short after anchoring (Fig. 4b).

### Assessment of outcomes

#### A) Primary outcome

The number of patients who underwent surgery for bowel obstruction caused by Petersen’s hernia within 3 years after laparoscopic or robotic gastrectomy. The results will be assessed by the PI and an independent biomedical statistician, who collectively compile data from each institution.

#### B) Secondary outcomes

The following secondary outcomes will be examined:

- Procedures time (minutes)
- The presence of bleeding during the mesentery suture
- Hospital stay (days)
- Occurrence of postoperative small bowel obstruction within 30 days after surgery
- Short-term complications within 30 days after surgery
- Occurrence of Petersen’s hernia in relation to the use of anti-adhesive agents and anastomosis methods
- Condition of the bowel (i.e., strangulation, perforation) at the time of emergency surgery for the treatment of Petersen’s hernias after primary surgery

### Data collection

Although all patients will be followed for 5 years postoperatively according to the cancer surveillance protocol, the primary endpoint is set to the results within 3 years after gastrectomy. Once the last participant reaches the 3-year follow-up observation, we will perform the final single analysis.

All data are recorded and managed accurately according to the appropriate format in the electronic case report form (eVelos system). The central research center evaluates the completeness and accuracy of the data collected and submitted at each center. Although the data collectors are not blinded, they do not influence patient enrollment or the intervention. The data collected are as follows:

#### A) Clinical observations

- Preoperative patient’s demographics and physical status: Age, sex, underlying disease, body mass index, ECOG score history of abdominal surgery, and clinical stage of gastric cancer
- Operative outcomes: Method of gastric resection, extent of lymph node dissection, reconstruction method, total operation time, CLOSURE or MEFIX procedure time, the presence of bleeding during the mesentery suture, and use of anti-adhesion agents during surgery
- Reoperation outcomes due to internal hernia: Type of herniation (Petersen’s or internal hernia), approach method (laparoscopic or open), condition of the bowel (i.e., strangulation, perforation), and site of herniated small bowel (E-loop or A-loop)
- Clinicopathologic and postoperative outcome: Pathologic TNM stage, first flatus time, time to start oral feeding, hospital stay, postoperative morbidity, and mortality.

#### B) Detailed complications that occur during the postoperative hospitalization period

- Short-term complications are defined within 30 days after surgery and are classified and recorded according to the Clavien–Dindo classification.
- Post-discharge investigations and long-term complications are as follows:
  Long-term complications are defined as those occurring 90 days postoperatively.
  Outpatient follow-up: Occurrence of Petersen’s hernia, other long-term complications, and incidence of readmission
  Occurrence of Petersen’s hernia: Petersen’s hernia requires reoperation.

The pathologic Petersen’s hernia is confirmed by surgical findings (i.e., the preoperative hernia is suspected, but hernias in other areas are diagnosed as internal hernias, not Petersen’s hernia). The clinical Petersen’s hernia before surgery is diagnosed through radiologic findings (mesenteric whirling sign, narrowing of superior mesenteric vein and dilated duodenum, etc.) with related symptoms such as nausea or vomiting. Abdominal CT is usually recommended for radiographic examinations. Ultrasonography is possible, but superior mesenteric vessel rotation must be confirmed.

### Data monitoring, auditing, and interim analysis

Researchers and CRC carry out the study at each institution. The entire research process is monitored by the Research Steering Committee, which is composed of the principal investigator (PI) and participating researchers with extensive research experience. Additionally, the Research Safety Monitoring Committee, independent from the study, was established to evaluate the safety of the research.

This study is conducted with respect for the rights and welfare of patients, and it is monitored whether the reported data related to the clinical research were accurate, complete, and verifiable compared with the evidence documents and whether the clinical research is conducted in accordance with the approved plans and relevant regulations. The patients’ original records, medical records, and data repositories (i.e., research files) will be reviewed every 6 months. All personal data will be stored in password-protected program; only authorized coordinator can access. The progress of clinical trial will be also reviewed, and any violations or noncompliance will be reported according to the institutional bioethics committee reporting procedure.

For reasons of safety and efficiency, interim analyses are planned to be conducted once 33% (first interim analysis) and 66% (second interim analysis) of the sample size have been recruited, and their results are available. The O'Brien-Fleming stopping rule will be applied to ensure efficacy and safety. If the primary outcome for the MEFIX group is superior to that of the CLOSURE group, with a *p*-value of less than 0.0005 at the first interim analysis, or less than 0.014 at the second interim analysis, consideration will be given to early termination of the study. This will allow for a prompt evaluation of the MEFIX method’s efficacy. In the final (third) analysis, we will not adjust the *p*-value to evaluate primary and secondary endpoints. There is no predefined stopping rule for safety related to complications. Any significant potential harm or serious adverse events (AEs) related to the procedures are considered grounds for stopping the trial.

This study is a prospective, multi-institutional clinical study. The steering committee will convene every 6 months to evaluate the progress and safety of the trial. Any safety concerns will be reported to the Research Safety Monitoring Committee, an independent organization from this trial, and assessed in the following cases.

1. The case of surgery due to Petersen’s hernia, which is the primary endpoint of this study, does not fall under the safety evaluation. During the interim analysis, if the upper margin of 9.7% of the incidence of Petersen’s hernia known through the existing references is exceeded, the research safety monitoring committee evaluates the safety of the study.
2. During the interim analysis, an evaluation will be conducted if there is a significant increase in complications suspected to be strongly related to the surgical method being performed in the experimental group.

### Adverse event

An adverse event (AE) refers to any undesirable medical occurrence in a patient that does not have a causal relation with the treatment. Therefore, an abnormal reaction is an unexpected and undesired symptom (for instance, abnormal laboratory test results) or a transient illness occurring independently of the surgery and without obvious causality. The severity of the AEs is assessed using the National Cancer Institute’s Common Terminology Criteria for Adverse Events (NCI-CTCAE), version 4.03, with detailed information being recorded in the patient’s medical record.

Serious AE is defined as a critical anomaly response if as follows: All deaths within 30 days of surgery are reported as significant AEs, regardless of the causality of the surgery. When a causal relationship is present in the study within 30 days of surgery, the following items are reported (i.e., causal relationships are reported separately as defined, probable, and passive): hospitalization due to severe complications or an extended hospitalization period after surgery. If a patient requires reoperation after the initial surgery (specifically, surgery necessitating general anesthesia in the operating room), any permanent or significant impairment related to the surgical procedure is reported. This includes brain lesions resulting in permanent aftereffects that are associated with the surgery.

If severe or medically significant clinical AEs or abnormal laboratory test results occur during or after the clinical trial period, the investigator shall report them to the PI and Clinical Trial Institutional Review Board (IRB) of the testing hospital, regardless of the treatment the patient received. The critical AEs are then reported by the PI to each responsible researcher at the participating institution, who subsequently reports them to the IRB of the affiliated institution.

### Ethics

Our study is conducted in accordance with the recent Declaration of Helsinki as amended by the 64th World Congress of Fortaleza, Brazil, 2013. In addition, clinical research plans and related documents are submitted to the institutional bioethics committee before clinical research is conducted in accordance with national laws and regulations. Clinical research is initiated after approval, and all individuals involve in the research below the researcher in charge adhered to good clinical practice (GCP). The study protocol was registered at ClinicalTrials.gov as NCT05105360.

## Discussion

Laparoscopic gastrectomy is considered the standard surgical method for EGC. With development of technology, patients’ recovery and quality of life have improved with minimally invasive surgery, such as total laparoscopic gastrectomy; however, the incidence of obstruction of the small intestine by internal hernias has increased. Minimally invasive procedures are thought to reduce the incidence of adhesions and cause Petersen’s hernia, which increases the incidence of small intestine obstruction with a frequency of 1.7–9.7%. To reduce this, Petersen’s and jejunostomy occlusions of the mesenteric defect have been performed, and several studies have shown that such an approach can reduce the incidence of internal hernias [10]. However, the commonly performed method of Petersen’s mesenteric defect closure prolongs the operative time and may cause ischemia of the small intestine due to damage to the mesenteric vessels and bleeding. It also increases the risk of early small bowel obstruction after surgery with kinking of the jejunojejunostomy [13]. Various methods have been introduced to prevent kinking of the jejunostomy, such as anti-obstructive suture [14], wide division of the mesentery, and double stapling of the jejunojejunostomy; however, the evidence is weak.

We have proposed the MEFIX method for fixing of the mesentery of the jejunum to the transverse mesocolon. This method has three advantages over the conventional methods. First, fixation of the mesentery located within 30 cm distal to the jejunojejunostomy to the mesentery of the transverse colon not only prevents excessive herniation of the small intestine due to a defect but also reduces the tension exerted on the jejunojejunostomy to prevent kinking. When the mesentery of the small intestine is anchored in the transverse colon between the small intestine and the mesentery of the colon, the defect remains. However, it prevents Petersen’s herniation by preventing total herniation of the mesentery of the small intestine even if partial internal hernia is allowed by producing the effect of adhesion of the small intestine to the surgical site during laparotomy. Second, the operation time can be shortened because the middle part is sutured instead of suturing continuously from the root to the peripheral part of the mesentery. Third, by suturing from the root artery to the arcade artery with relatively few blood vessels without suturing to the vasa recta vessels of the small intestine, complications such as bleeding and ischemia can be reduced by decreasing vascular damage.

## Conclusions

This study is a prospective, randomized controlled trial to compare the MEFIX and CLOSURE methods, intending to confirm that the MEFIX method is not inferior to the CLOSURE method. If the results are positive, the MEFIX method could prevent Petersen’s hernia and reduce operation time and complications. Moreover, the MEFIX method is a new concept and technique that have not been previously reported. It is anticipated to be applicable not only in gastric cancer surgery but also in bariatric surgery, especially in Roux-en-Y gastric bypass procedures.

### Trial status

This trial was registered at ClinicalTrials.gov as NCT05105360 on November 3, 2021. The first patient was enrolled on April 18, 2022. At the time of submitting this protocol for publication, all centers were actively recruiting patients for the trial, and 159 out of 426 (37%) have been enrolled.

### Supplementary Information

**Additional file 1.** SPIRIT Checklist for Trials.

## Data Availability

Not applicable.

## Abbreviations

AE: Adverse events
AGC: Advanced gastric cancer
B-II: Billroth-II
CT: Computed tomography
ECOG: Eastern Cooperative Oncology Group
EGC: Early gastric cancer
GCP: Good clinical practice
GJ: Gastrojejunostomy
IRB: Institutional review board
MEFIX: Mesentery fixation
NCI-CTCAE: National Cancer Institute Common Terminology Criteria for Adverse Events
PI: Principal investigator
REY: Roux-en-Y
SB: Small bowel
